# β3-Adrenoreceptor Blockade Reduces Hypoxic Myeloid Leukemic Cells Survival and Chemoresistance

**DOI:** 10.3390/ijms21124210

**Published:** 2020-06-12

**Authors:** Maura Calvani, Annalisa Dabraio, Gennaro Bruno, Veronica De Gregorio, Marcella Coronnello, Costanza Bogani, Sara Ciullini, Giancarlo la Marca, Marina Vignoli, Paola Chiarugi, Margherita Nardi, Alessandro Maria Vannucchi, Luca Filippi, Claudio Favre

**Affiliations:** 1 Division of Pediatric Oncology/Hematology, Meyer University Children’s Hospital, 50139 Florence, Italy; maura.calvani@meyer.it (M.C.); annalisa.dabraio@unifi.it (A.D.); gennaro.bruno@unifi.it (G.B.); veronica.degregorio@meyer.it (V.D.G.); sciulliniman@unifi.it (S.C.); marina.vignoli@unifi.it (M.V.); 2 Department of Health Sciences, University of Florence, 50139 Florence, Italy; marcella.coronnello@unifi.it; 3 Department of Experimental and Clinical Medicine, University of Florence, 50139 Florence, Italy; bogani.costanza@gmail.com (C.B.); amvannucchi@unifi.it (A.M.V.); 4 Department of Experimental and Clinical Biomedical Sciences, University of Florence, 50134 Florence, Italy; giancarlo.lamarca@unifi.it (G.l.M.); paola.chiarugi@unifi.it (P.C.); 5 Onco-Hematologic Pediatric Center, University Hospital of Pisa, 56126 Pisa, Italy; m.nardi@med.unipi.it; 6 Neonatal Intensive Care Unit, Medical Surgical Fetal-Neonatal Department, Meyer University Children’s Hospital, 50139 Florence, Italy; luca.filippi@meyer.it

**Keywords:** chemoresistance, myeloid leukemia, β3-adrenoreceptor

## Abstract

β-adrenergic signaling is known to be involved in cancer progression; in particular, beta3-adrenoreceptor (β3-AR) is associated with different tumor conditions. Currently, there are few data concerning β3-AR in myeloid malignancies. Here, we evaluated β3-AR in myeloid leukemia cell lines and the effect of β3-AR antagonist SR59230A. In addition, we investigated the potential role of β3-AR blockade in doxorubicin resistance. Using flow cytometry, we assessed cell death in different in vitro myeloid leukemia cell lines (K562, KCL22, HEL, HL60) treated with SR59230A in hypoxia and normoxia; furthermore, we analyzed β3-AR expression. We used healthy bone marrow cells (BMCs), peripheral blood mononuclear cells (PBMCs) and cord blood as control samples. Finally, we evaluated the effect of SR59230A plus doxorubicin on K562 and K562/DOX cell lines; K562/DOX cells are resistant to doxorubicin and show P-glycoprotein (P-gp) overexpression. We found that SR59230A increased cancer cell lines apoptosis especially in hypoxia, resulting in selective activity for cancer cells; moreover, β3-AR expression was higher in malignancies, particularly under hypoxic condition. Finally, we observed that SR59230A plus doxorubicin increased doxorubicin resistance reversion mainly in hypoxia, probably acting on P-gp. Together, these data point to β3-AR as a new target and β3-AR blockade as a potential approach in myeloid leukemias.

## 1. Introduction

Leukemia is the term used to identify a group of different cancers involving blood and bone marrow (BM). Myeloid malignancies are clonal disorders associated with uncontrolled proliferation and altered differentiation of hematopoietic stem cells (HSCs) with a consequent increase in immature myeloid cells [1,2]. 

In recent years, the development of new technologies and the discovery of novel molecular findings, have improved leukemia diagnosis. An important challenge in the treatment of myeloid malignancies is represented by the phenomenon of resistance to antineoplastic drugs. Understanding the cellular and molecular mechanisms associated with chemoresistance is crucial in order to improve cancer patients’ outcome and survival. 

There are two main types of chemoresistance: primary drug resistance and acquired drug resistance. In patients with primary resistance, tumor cells appear resistant before chemotherapy; while in patients with acquired resistance, tumor cells become resistant after antineoplastic treatment [3]. In general, drug resistance is the result of a combination of multiple factors and biological mechanisms among which altered expression and activity of specific proteins, genetic and epigenetic changes, deregulation of signal transduction pathways [3,4]. 

It is known that an abnormal expression of efflux proteins, and especially of P-glycoprotein (P-gp), is associated with chemoresistance in myeloid neoplasms [4,5,6]. P-gp is an ATP-binding Cassette (ABC) transporter encoded by the multiple drug resistance (*MDR1)* gene. In particular, it is a 170-kDa efflux pump, which using ATP hydrolysis, plays an important role in the extrusion of different compounds out of cells, including drugs and xenobiotics, with a consequent decrease in intracellular substances accumulation. P-gp is expressed in healthy tissues but also in different types of cancer [7]. Interestingly, P-gp overexpression in tumors, including myeloid neoplasms [4,5], enhances drugs extrusion out of cells, reducing chemotherapy efficiency and promoting the phenomenon of resistance to multiple antineoplastic agents [7]. For instance, an association of a high level of P-gp with a poor outcome is known in acute myeloid leukemia (AML) [4]. Moreover, Schaich et al., reported that *MDR1* expression was an independent prognostic factor for induction therapy outcome and overall survival in AML patients [8]. 

βeta-adrenergic receptors (β-ARs) are G-protein-coupled receptors involved in catecholamines-activated signal transduction pathways. Three types of β-ARs are known: beta1-adrenoreceptors (β1-ARs), beta2-adrenoreceptors (β2-ARs) and beta3-adrenoreceptors (β3-Ars). These receptors are localized and expressed in distinct and specific tissues. β1-ARs are expressed abundantly in cardiac tissue, kidney and adipose tissue; β2-ARs are localized in gastrointestinal tract, bronchi, skeletal muscle, liver, immune and non- immune cells; finally, β3-ARs are mainly present in intestine, adipose tissue and endothelium, moreover they are expressed in the smooth muscle cells of the detrusor muscle in the urinary bladder [9]. Interestingly, β3-ARs expression is reported also in Chinese hamster ovary/K1 cells [10]. β-ARs are involved in the modulation of different physiological processes, such as metabolism and cardiovascular function, but also in human diseases, including cancer [9,11]. Indeed, several studies have described β-ARs expression in various tumor types and especially in melanoma, vascular tumors and lung, pancreatic, colorectal, brain, breast, ovarian, prostate, hepatic, kidney and adrenal cancer [9,11]. Interestingly, β3-ARs expression has been reported also in human leukemia cells [12].

β-ARs play a key role in different biological processes that are crucial in cancer biology and they promote tumor progression [13]. In particular, β-ARs are involved in inflammation, angiogenesis, cancer cells migration, proliferation and survival, epithelial-mesenchymal transition, invasiveness, metastasis, apoptosis, cellular immune response and resistance to chemotherapy-induced apoptosis [9,13]. Among the β-ARs, the β2-AR subtype has been shown to be involved in biological processes related to cancer [14]; however, in recent years, the role of β3-AR in the regulation of cancer-related pathways has emerging in different types of cancer, especially in melanoma [15].

Furthermore, β-ARs expression has been showed not only in cancer cells, but also in tumor microenvironment cells, including cancer associated fibroblasts, macrophages, and endothelial cells [11,13]. 

Finally, different studies suggest that β-AR blocker drugs are associated with reduction of cancer cell proliferation, progression and metastasis improving outcome and survival [9,11]. For instance, β3-AR antagonist SR59230A promotes tumor cells death and reduces angiogenesis and proliferation in melanoma [9,16].

In this study, we investigated the effect of β3-AR antagonist SR59230A, belonging to the class of aryloxypropanolaminotetralins, on different in vitro models of myeloid leukemias. Moreover, we analyzed the potential involvement of β3-AR in the phenomenon of chemoresistance, which generally represents a crucial challenge in cancer treatment. Indeed, chemoresistance influences patients’ clinical outcome promoting recurrence and metastasis and increasing mortality risk.

Here, we demonstrate in in vitro models that β3-AR is highly expressed in myeloid malignancies and could be involved in cancer cell lines survival in particular under hypoxic conditions; in addition, we show that SR59230A treatment in combination with doxorubicin could reduce resistance to doxorubicin, especially in hypoxia.

## 2. Results

### 2.1. SR59230A Promoted Apoptosis of Leukemia Cell Lines Preferentially in Hypoxia

**Figure 1:**
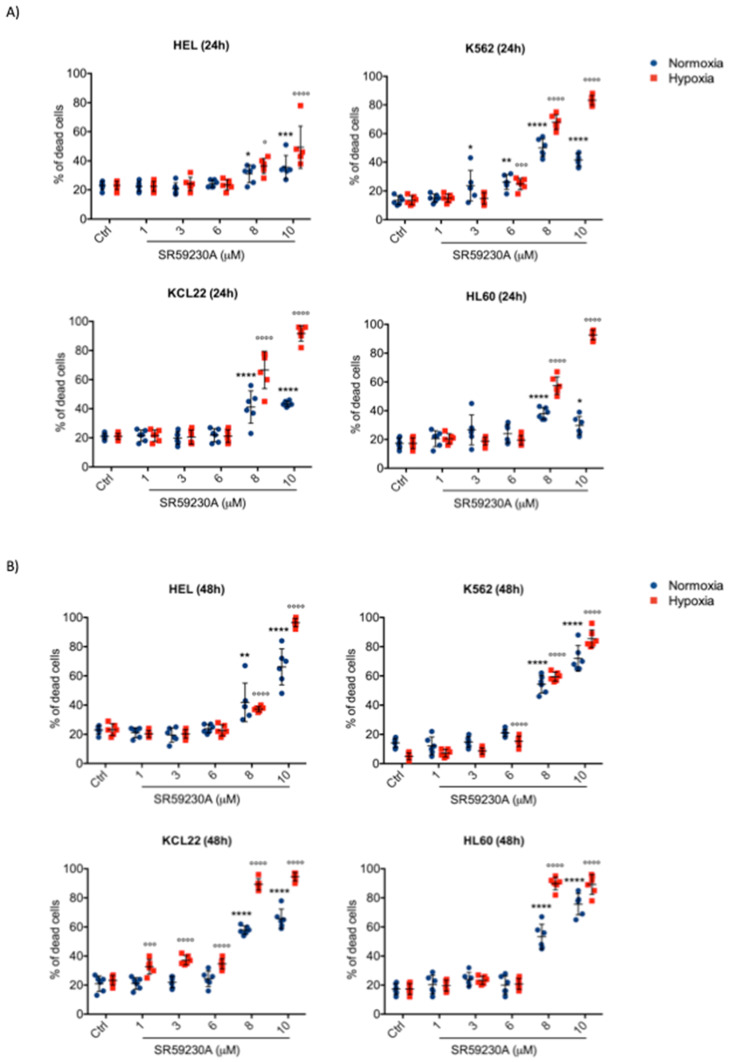
Cell death assessment in myeloid leukemia cell lines treated with SR59230A in hypoxia (1% O_2_) and in normoxia (21% O_2_). (**A**) Apoptosis evaluation through Annexin V and Propidium Iodide in HEL, K562, KCL22 and HL60 cell lines treated with different concentrations of SR59230A (1 μM, 3 μM, 6 μM, 8 μM, 10 μM) for 24 h, in normoxia and in hypoxia; (**B**) apoptosis evaluation through Annexin V and Propidium Iodide in HEL, K562, KCL22 and HL60 cell lines treated with different concentrations of SR59230A (1 μM, 3 μM, 6 μM, 8 μM, 10 μM) for 48 h, in normoxia and in hypoxia. Significance was calculated by one-way ANOVA analysis followed by Bonferroni’s post-hoc test. Results are reported as mean ± SD of three independent experiments performed in duplicate. n = 6 per group. (* *P* < 0.05, ** *P* < 0.01, *** *P* < 0.001, **** *P* < 0.0001 SR vs. Ctrl normoxia; ° *P* < 0.05, °°° *P* < 0.001, °°°° *P* < 0.0001 SR vs. Ctrl hypoxia).

In order to investigate the effect of β3-ARs blockade on apoptosis in leukemic malignancies, four different cell lines of myeloid leukemia were treated with β3-ARs antagonist SR59230A (Figure 1). SR59230A effect was analyzed in K562, KCL22, HEL and HL60 cell lines, using different drug concentrations (1 μM, 3 μM, 6 μM, 8 μM, 10 μM) in hypoxic and normoxic conditions, 24 h and 48 h after drug treatment. 

As reported in Figure 1, SR59230A increased apoptosis with a dose-dependent modality in all cell lines with maximal effects at 8 μM and 10 μM. Moreover, an enhancement of cell lines death was observed in hypoxia and especially after longer drug exposure, 48 h; no relevant differences were observed between 24 h and 48 h treatment in normoxia. After 48 h of treatment most of the cell lines treated underwent apoptosis (ranging from 80% to 95%) (Figure 1B). To better discriminate the role played by β3-AR, we treated three different myeloid leukemia cell lines (HEL, HL60, K562) with another β3-adrenoceptor antagonist, L748,337, and with non-selective β1/β2-AR antagonist propranolol. Results are reported in Supplementary Figure S2A. L 748,337 inhibited all three cell lines at both concentrations (5 μM and 10 μM), conversely, propranolol showed a less marked effect on the inhibition of the three cell lines survival. Moreover, to discriminate the role of β3-AR in the effect observed following SR59230A and L748,337 treatment, we used selective siRNAs for β1-, β2-, β3-ARs (Supplementary Figure S1A). These results suggested the predominant role of β3-AR subtypes in the regulation of cell survival in these leukemia cell lines.

### 2.2. β3-AR Expression Increased under Hypoxia in Myeloid Leukemia Cell Lines

It is known that β3-AR is highly expressed in different tumor tissues, including hematologic malignancy [17]. Therefore, we investigated the β3-AR expression in myeloid leukemia cell lines used in this analysis under normoxia and hypoxia conditions. 

**Figure 2:**
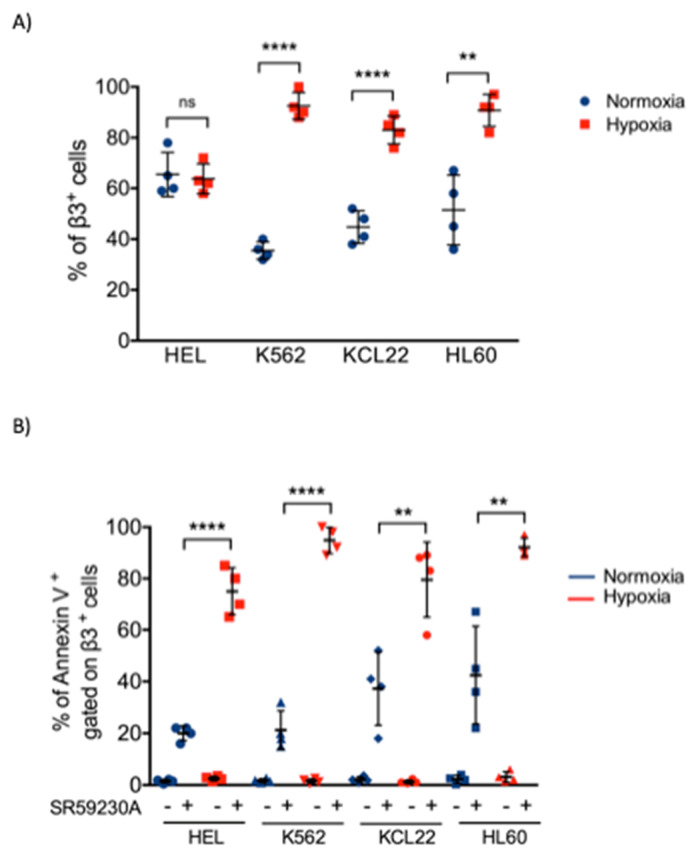
Evaluation of beta3-adrenoreceptor (β3-AR)-positive cells and β3-AR/Annexin V-positive cells in myeloid leukemia cell lines under hypoxia (1% O_2_) and normoxia (21% O_2_) for 48 h. (**A**) β3-AR-positive cells in HEL, K562, KCL22 and HL60 cell lines in normoxia and hypoxia; significance was calculated by unpaired T-test in each cell line. Results are reported as mean ± SD of four independent experiments. n = 4 per group (** *P* < 0.01, **** *P* < 0.0001 hypoxia vs. normoxia). (**B**) Flow cytometric analysis of HEL, K562, KCL22 and HL60 cell lines in control and SR59230A 5 µM treated cells in normoxia and hypoxia. Annexin V-positive cells gated on a β3-AR-positive cells subpopulation are showed. Significance was calculated by unpaired T-test in each cell lines. Results are reported as mean ± SD of four independent experiments. n = 4 per group. (** *P* < 0.01, **** *P* < 0.0001 SR hypoxia vs. SR normoxia).

Notably, data revealed an increase in β3-AR expression in K562, HL60 and KCL22 cell lines after 48 h of hypoxic exposure (Figure 2A), while the HEL cell line showed a different behavior. On the contrary, neither β1-AR or β2-AR protein expression resulted upregulated under hypoxic conditions (Supplementary Figure S2B). Interestingly, we detected that a high percentage of β3-AR-positive cells were also Annexin V-positive, especially in hypoxic conditions after SR59230A treatment, demonstrating an enhancement of apoptosis in β3-AR-positive cells. Since the most of β3-AR-positive cells were apoptotic, this suggested a selective activity of the treatment with SR59230A on leukemia cell lines (Figure 2B). 

### 2.3. SR59230A Was not Toxic in Healthy Cell Lines 

To exclude drug toxicity, we performed an analysis of SR59230A effect on bone marrow cells (BMCs) samples of healthy donors; in particular, BMCs were treated under normoxia and hypoxia conditions for 48 h. 

**Figure 3:**
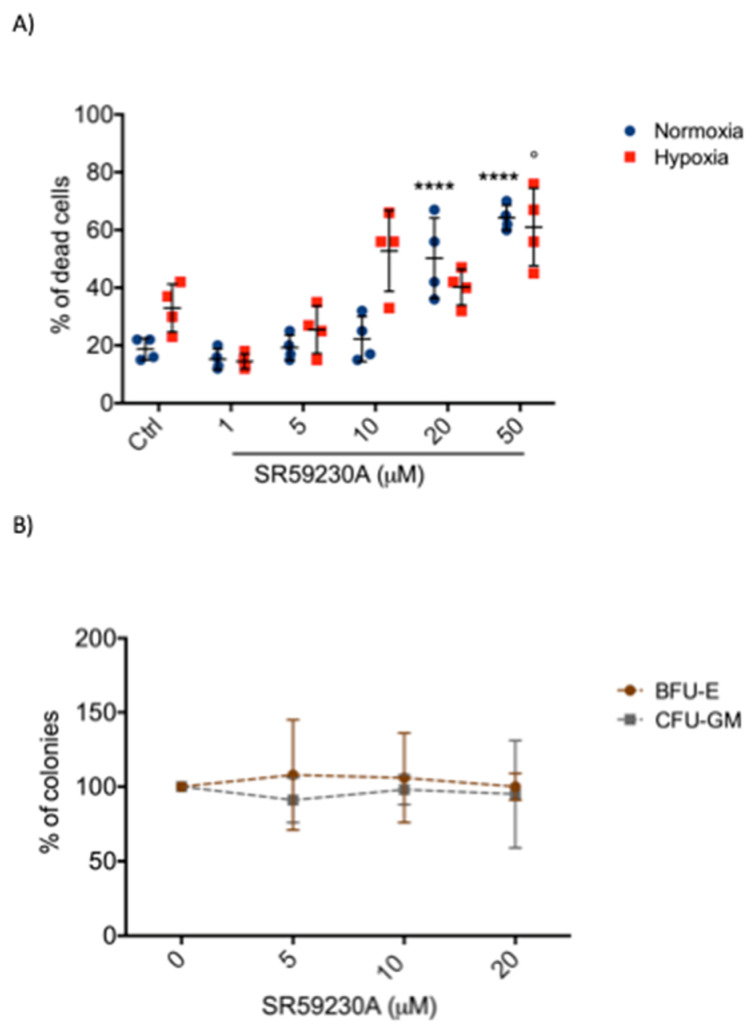
Evaluation of SR59230A effect on bone marrow cells (BMCs) and cord blood samples of healthy donors. (**A**) Apoptosis evaluation in BMCs samples treated with different concentrations of SR59230A (1 μM, 5 μM, 10 μM, 20 μM, 50 μM) in normoxia (21% O_2_) and hypoxia (1% O_2_); significance was calculated by one-way ANOVA analysis followed by Bonferroni’s post-hoc test. Results are reported as mean ± SD of four independent experiments. n = 4 per group. (**** *P* < 0.0001 SR vs. Ctrl normoxia; ° *P* < 0.05 SR vs. Ctrl hypoxia). (**B**) SR59230A (5 μM, 10 μM, 20 μM) effect on colony formation (BFU-E = burst forming units-erythroid; CFU-GM = colony formation units-granulocyte, monocyte) in cord blood samples donor. Results are reported as mean ± SD of three independent experiments. n = 3 per group.

As shown in Figure 3A, BMCs displayed low sensitivity to SR59230A in comparison to leukemic cell lines, suggesting a possible involvement of β3-AR in cancer cells. Particularly, we observed a relevant BMCs death only at high dose of SR59230A, in a range concentration between 10 and 50 μM. Moreover, the effect of SR59230A on the colony formation was analyzed in healthy cord blood donor. The presence of SR59230A did not statistically change the number of different colonies (Figure 3B). In the SR59230A groups, under normoxic conditions the number of colony formation units-granulocyte, monocyte (CFU-GM) and burst forming units-erythroid (BFU-E) remained almost stable in all concentrations used.

### 2.4. β3-AR Was Expressed at Low Levels in Healthy Cells

**Figure 4:**
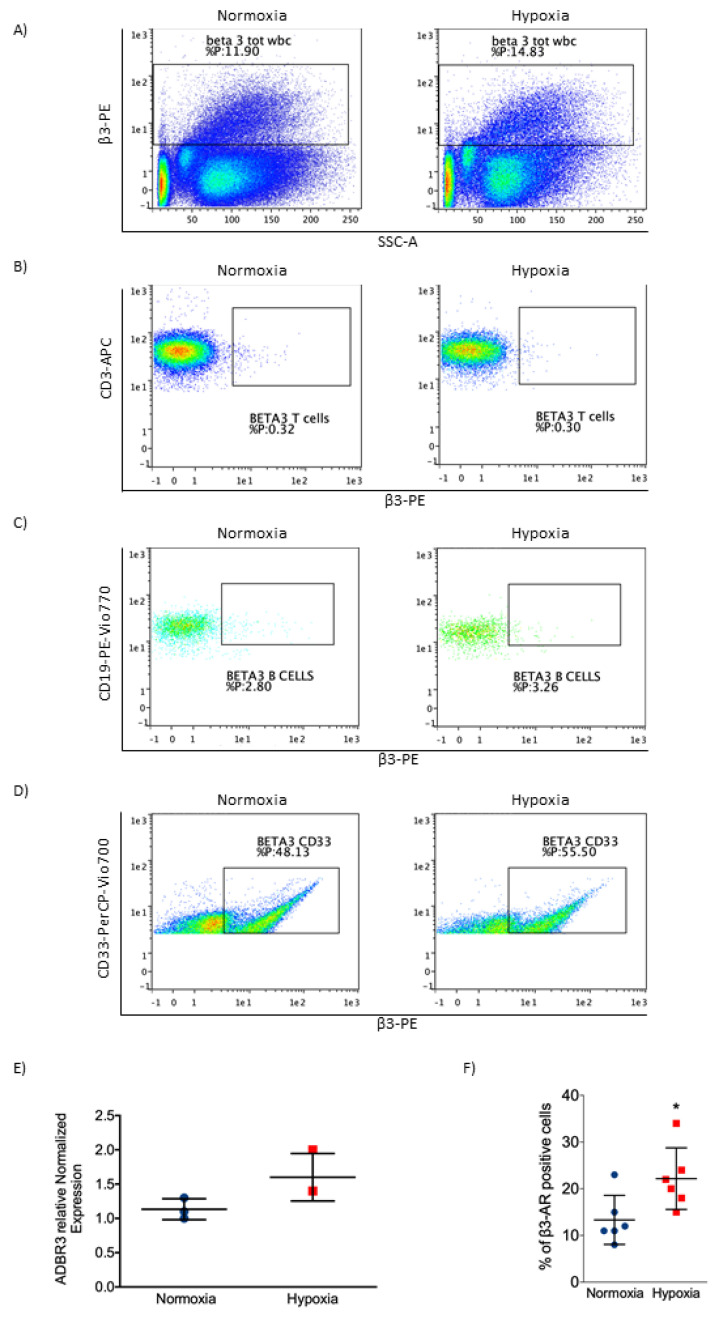
Evaluation of β3-AR expression in BMCs and peripheral blood mononuclear cells (PBMCs) of healthy samples. (**A**) Flow cytometric plot showing β3-AR expression on total PBMCs. (**B**) Flow cytometric plot showing β3-AR expression on CD3 (T cells) positive cells. (**C**) Flow cytometric plot showing β3-AR expression on CD19 (B cells) positive cells. (**D**) Flow cytometric plot showing β3-AR expression on CD33 (myeloid cells) positive cells. (**E**) Real-time PCR showing β3-AR mRNA expression in PBMCs in normoxia (21% O_2_) and hypoxia (1% O_2_). (**F**) β3-AR positive cells in BMCs under normoxia and hypoxia, evaluated through cytofluorimetric analysis. Results are reported as mean ± SD of three independent experiments. n = 3 per group. * *P* < 0.05 hypoxia vs. normoxia.

To clarify the ineffectiveness on healthy samples we analyzed the expression of β3-AR in healthy cells such as BMCs and peripheral blood mononuclear cells (PBMCs). As reported in Figure 4A–D, the expression of β3-AR is low in healthy PBMCs and BMCs. Under hypoxic condition, β3-AR resulted slightly upregulated in PBMCs. Among the different subtypes of PBMCs, β3-AR resulted in a greater expression in myeloid lineage (Figure 4D). Real time PCR confirmed the low level of β3-AR in normoxia and a slight induction under hypoxic condition in PBMCs (Figure 4E); moreover, through cytofluorimetric analysis, we observed an increase in β3-AR positive cells in BMCs under hypoxic conditions (Figure 4F). All these results supported the hypothesis that β3-AR is strongly upregulated in myeloid leukemia cell lines and that it could participate in maintaining cell survival in this pathological condition. Our results showed that β3-AR expression increased in cancer, suggesting a potential role of this protein in cancer biology.

### 2.5. K562 Doxorubicin Resistant Cell Line and SR59230A Treatment 

**Figure 5:**
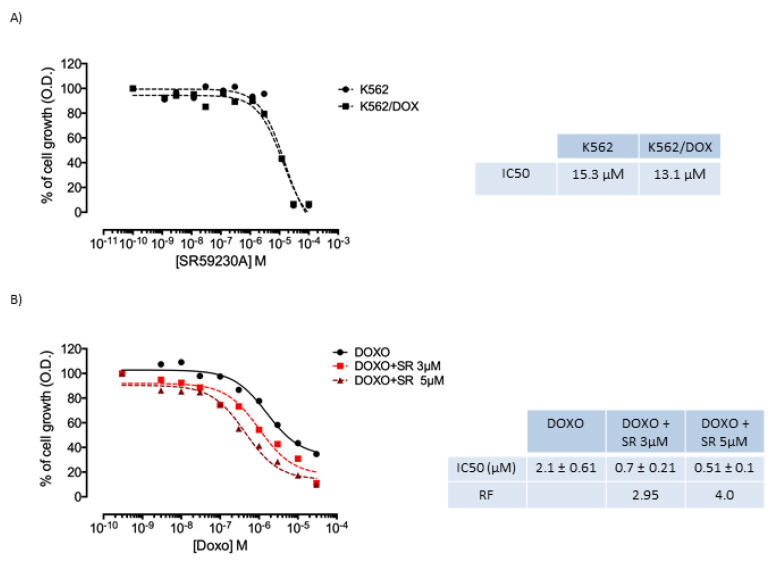
Evaluation of cytotoxicity of SR59230A and SR59230A/doxorubicin combination. (**A**) Intrinsic cytotoxicity of SR59230A evaluated on K562 cell line and K562/DOX cell line resistant to doxorubicin due to P-glycoprotein (P-gp) overexpression. The table shows IC_50_ values obtained with SR59230A; (**B**) Cytotoxicity curves of doxorubicin in the absence and in the presence of two concentrations of SR59230A (3 µM and 5 µM) in K562/DOX cell line. Average IC_50_ values ± error standard of doxorubicin and its combination with the SR59230A are reported. The table also shows the reversal fold (RF) value calculated from the ratio between the IC_50_ value of doxorubicin in the absence of SR59230A and in the combination with the compound under study. Results are reported as mean of three independent experiments.

Since SR59230A was very effective in inducing apoptosis in our experimental setting, we evaluated whether SR59230A could sensitize the doxorubicin resistant K562/DOX cell line. SR59230A showed half maximal inhibitory concentration (IC_50_) values which for K562 and K562/DOX cell lines were 15.3 and 13.1 µM, respectively. Therefore, the two lines had the same sensitivity towards the compound. These results led us to continue the studies with two concentrations, 3 µM and 5 µM, which had an intrinsic toxicity of around 20% (Figure 5A).

As shown in Figure 5B, SR59230A seemed to increase the sensitivity of the K562/DOX cell line to doxorubicin, producing a reversal fold (RF) of 2.95 with the 3 µM concentration and of 4 with the 5 µM concentration under normoxic condition. The RF values are derived from a reduction in the IC_50_ values for doxorubicin which stand at 2.1 ± 0.61 µM, 0.7 ± 0.21 µM and 0.51 ± 0.1 µM for anthracycline in the absence of SR59230A and in the presence of SR59230A (3 µM and 5 µM, respectively).

**Figure 6:**
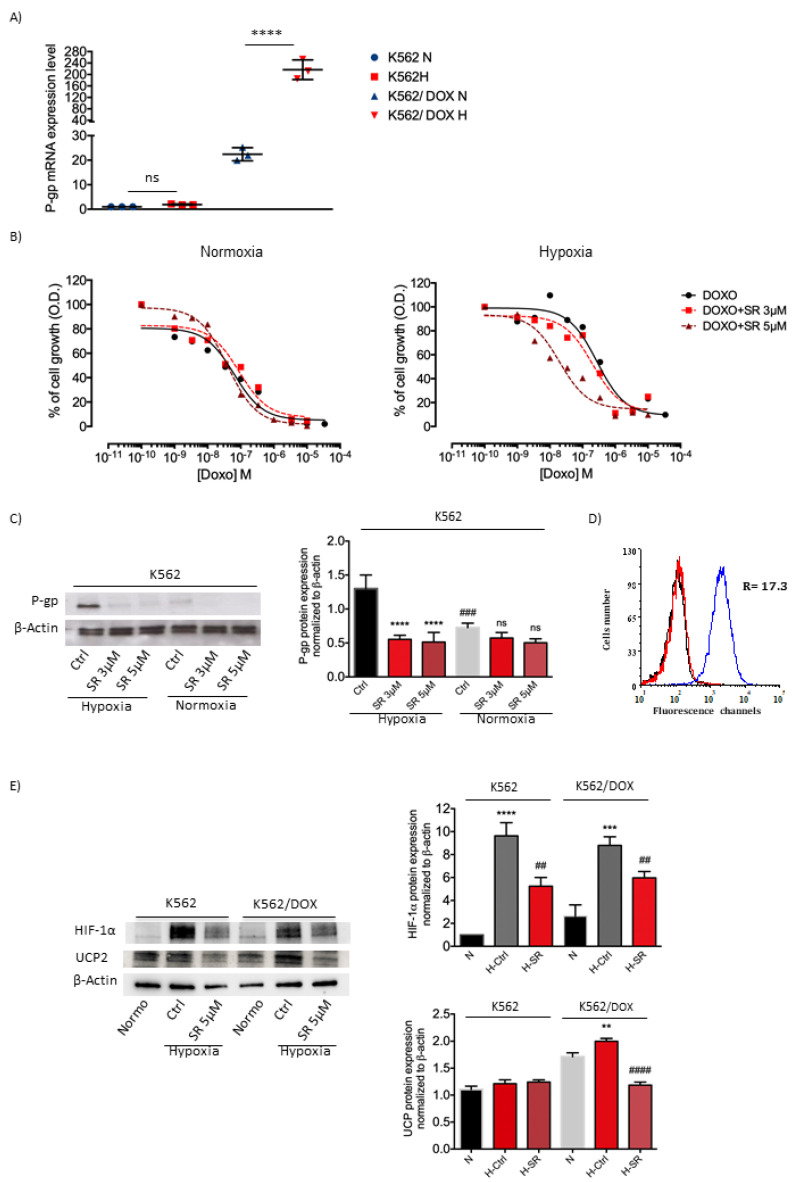
P-gp, HIF-1α and UCP2 expression, and evaluation of cytotoxicity of SR59230A and SR59230A/doxorubicin combination. (**A**) Real-time PCR analysis: P-gp mRNA expression assessed in K562 and K562/DOX cell lines under hypoxia (1% O_2_) and normoxia (21% O_2_); significance was calculated by one-way ANOVA analysis followed by Bonferroni’s post-hoc test. Results are reported as mean ± SD of three independent experiments. n=3 per group. (**B**) Cytotoxicity curves of doxorubicin in normoxia and hypoxia in the absence and in the presence of two concentrations of SR59230A (3 µM and 5 µM) in K562 cell line. Results are reported as mean of three independent experiments. (**C**) Western blot analysis showing P-gp expression in K562 cells treated with SR59230A at 3 µM and 5 µM under hypoxia and normoxia. Significance was calculated by one-way ANOVA analysis followed by Bonferroni’s post-hoc test. Results are reported as mean ± SD of three independent experiments. n = 3 per group. (**D**) Fluorescence curves obtained with a FACScanto flow cytometer. R= ratio between the mean fluorescence intensity of resistant cells and parental cells. K562 cells (red) and K562/DOXO cells (blue), black curve: autofluorescence. (**E)** Western blot analysis showing HIF-1α and UCP2 expression in K562 and K562/DOXO cells treated with SR59230A at 5 µM under hypoxia and relative control in normoxia. Significance was calculated by one-way ANOVA analysis followed by Bonferroni’s post-hoc test. Results are reported as mean ± SD of three independent experiments. n = 3 per group.

A preliminary study was conducted on the K562 cell line to investigate a putative role of the β3-AR in multidrug resistance (MDR). According to literature [18], the hypoxic environment should increase the expression of P-gp; therefore, the response of the K562 and K562/DOX cell lines to hypoxic exposure was assessed. As shown in Figure 6A, K562 cell line in a hypoxic environment moderately increased the expression of P-gp compared with the same condition in K562/DOX cell line. K562/DOX cell line revealed higher expression of P-gp and a strong induction under hypoxic conditions. Cytotoxicity of doxorubicin increased significantly with RF of 14.2 in K562 cell line exposed to hypoxia (Figure 6B and Supplementary Figure S1B). Moreover, results showed that SR59230A decreased P-gp protein expression both at 3 µM and 5 µM under hypoxic conditions (Figure 6C). Figure 6D shows the fluorescence curves obtained with the CD243 antibody specific for P-gp; the figure shows that the protein is overexpressed in the resistant cell line with a fluorescence ratio of 17.3. 

According to Calvani et al., UCP-2 is strongly expressed in various cancer types, among which leukemia and pancreatic cancer. Moreover, UCP-2 overexpression is regulated by β3-ARs in stem cells [15]. Furthermore, literature data showed that UCP-2 is involved also in cancer resistance, especially in paclitaxel resistance of lung cancer, in topoisomerase I inhibitor CPT-11 resistance of colon cancer and in gemcitabine resistance of pancreatic adenocarcinoma, non-small cell lung adenocarcinoma and bladder carcinoma [19]. Interestingly, it has been also reported in leukemia that genipin, an UCP-2 inhibitor, sensitized drug-resistant cells to anthracyclin [20].

Therefore, in this report, we treated K562 both sensitive and resistant to doxorubicin with SR59230A. Results showed that, in K562 resistant cell line, SR59230A inhibited the upregulation of UCP2 observed in hypoxia compared to normoxia conditions (Figure 6E). 

Moreover, we observed also that HIF-1a protein expression induced in hypoxia was reverted by treatment with SR59230A (Figure 6E), confirming that the β3-AR could affect MDR-1 expression by regulating the HIF-1a protein levels. 

## 3. Discussion

Literature data showed that β-ARs blockade could be involved in prevention and treatment of different types of tumor [21]. Preclinical and clinical efficacy of β-ARs blockade in numerous cancers, including breast cancer, melanoma, angiosarcoma, neuroblastoma, pancreatic adenocarcinoma, ovarian and prostate cancer, has been demonstrated [22]. For instance, Montoya et al. showed that β-ARs antagonists may promote a reduction of tumor proliferation not only in localized breast tumors [23], but also in advanced stages [22]. Concerning blood cancer, Lamkin et al. showed through in vivo models that chronic stress can enhance the progression of human pre-B cell acute lymphoblastic leukemia involving β-adrenergic signaling [12]. Nevertheless, currently the role of β-AR antagonists in myeloid malignancies has not been clarified yet.

In order to better understand the role of β3-AR in myeloid malignancies, we focused our attention on the effect of the β3-AR antagonist SR59230A on this type of tumor; in particular, we used K562, KCL22, HEL and HL60 myeloid leukemia cell lines and we demonstrated that β3-AR blockade results in reduced cancer cell lines survival by increasing apoptosis, especially under hypoxic conditions.

The finding that SR59230A enhances cell lines death supports the idea that β3-AR could influence biological processes involved in the regulation of the balance between cell growth and apoptosis. To confirm that β3-AR could play a crucial role in disease condition, and especially in cancer, we analyzed BM and PBMCs samples from healthy donors as control samples. SR59230A was selective for myeloid leukemia malignancies and non-toxic for normal cells, demonstrating that β3-AR could be a possible marker for malignancies. 

Literature data have already shown the β3-AR upregulation under hypoxia in melanoma [16]; here we demonstrated that β3-AR expression is increased in myeloid leukemia cell lines under hypoxia. Moreover, we observed an enhancement of apoptosis in β3-AR positive cells after SR59230A treatment, suggesting that β3-AR could contribute to cancer cell lines survival. Our results suggest to better investigate the efficacy of β3-AR blockers as potential drugs for myeloid leukemias treatment. β3-AR is expressed in normal cells at low levels and more importantly is slightly induced by hypoxia compared with hypoxic induction in cancer cell lines. The expression of β3-AR in normal cells, in particular in BM, was reported by Méndez-Ferrer et al. showing the participation of the receptor in HSCs mobilization from the BM [24]. In particular, the authors discovered that adrenergic hormones, known for their involvement in bone remodeling binding β2-ARs on osteoblasts, were also involved in the activation of β3-ARs on the surface of BM stromal cells with consequent degradation of the Sp1 transcription factor, downregulation of the anchoring protein CXCL12 and finally HSCs mobilization from BM to bloodstream [24,25].

Since β3-AR was slightly induced under hypoxic conditions in normal cells and hypoxia is a feature of different pathologies, this receptor could be a putative selective marker for cancer therapy and other pathologies involving hypoxic environment.

Furthermore, in this study we focused our attention on the potential effect of the β3-AR antagonist SR59230A on myeloid leukemia’s resistance to doxorubicin. Indeed, cancer MDR represents a significant clinical problem for cancer treatment, including hematological malignancies. Doxorubicin is an antineoplastic compound frequently used in different tumor types [26]; unfortunately, myeloid leukemia treatment often fails due to the development of resistance to doxorubicin. In general, MDR is a complex process associated with various mechanisms, including overexpression of ABC transporters, among which is P-gp, with consequent increase in drugs efflux [27].

Several studies evidenced the involvement of HIF1α in the regulation of *MDR1* gene expression in various tumors, including colon and liver cancer [28]. In particular, hypoxia promotes chemoresistance by enhancing *MDR1* expression in tumor cells [28]; for instance, Xie et al. reported a correlation of HIF1α expression and MDR1/Pgp expression in laryngeal cancer Hep 2 cells [28], while Ding et al. described this association in colon carcinoma [29]. Moreover, the relationship between HIF-1, hypoxia and P-gp has been described also in gastric cancer, gliomas and breast carcinoma [30]. Concerning hematologic malignancy, interestingly Muz et al. reported that hypoxia increased P-gp expression in an in vitro model of multiple myeloma [31].

In this work we showed that the combination of β3-AR antagonist SR59230A with doxorubicin reduced resistance to doxorubicin in K562/DOX cell line, which represents a model of a doxorubicin-resistant cell line with higher expression of P-gp in comparison with the K562 cell line. 

We showed an increase in *MDR1* expression in the K562 cell line maintained in hypoxia, consistent with the finding that hypoxia is involved in MDR [32]; we then evaluated the effect of SR59230A and doxorubicin combination on this parental cell line under normoxic and hypoxic conditions observing an increase in doxorubicin sensitivity in hypoxia in comparison with normoxia. 

According to Comerford et al., *MDR1* gene is hypoxia responsive: indeed, hypoxia promotes transcriptional induction of *MDR1* and consequently P-gp expression [18]. Finally, the resistance reversion in K562 resistant to doxorubicin cell line treated with SR59230A suggests that SR59230A could act targeting P-gp or could be involved in the regulation of P-gp expression. We can therefore speculate that SR59230A may represent a novel interesting therapeutic strategy for myeloid leukemias treatment resistant to MDR drugs.

## 4. Materials and Methods 

### 4.1. Cell Lines and Culture Conditions

Four different human myeloid leukemia cell lines were cultured in our study: K562 and KCL22 (chronic myelogenous leukemia cell lines), HEL (erythroleukemia cell line), HL60 (Acute Promyelocytic Leukemia cell line). Cell lines were obtained from ATCC. Cell lines were cultured in RPMI medium (Gibco) supplemented with 10% fetal bovine serum (FBS), 1% L-glutamine (200 mmol/L), 100 U/mL penicillin–streptomycin. All cell lines were maintained at 37 °C in a 5% CO_2_ humidified atmosphere incubator with 21% O_2_ for normoxia, and 1% O_2_ for hypoxia condition. 

The P-gp expressing K562/DOX cell line was obtained from Prof. J. P. Marie (Hospital Hotel-Dieu, Paris, France). This cell line was cultured in RPMI 1640 medium (GIBCO) supplemented with 10% fetal calf serum (FCS) (GIBCO) at 37 °C in a humidified incubator with 5% CO_2_. To maintain the resistance, every month, the resistant cell line was cultured for three days with 400 nM doxorubicin. K562/DOX cell line overexpresses almost exclusively the membrane glycoprotein P-gp. 

All cell lines were routinely tested for mycoplasma contamination.

### 4.2. Intrinsic Cytotoxicity 

The intrinsic toxicity of the SR59230A compound was determined through MTT assay after the exposure of parental cell line, K562, and resistant cell line, K562/DOX, to the compound in a concentration range of 10^10^ M to 10^4^ M for 72 h in a humidified atmosphere with 5% CO_2_. The MTT working solution was then added and plates were further incubated for 3 h. Following incubation cells, formazan crystals were inspected microscopically. The supernatant was then carefully removed by slow aspiration and the formazan crystals were dissolved in 150 μL of Dimethyl Sulfoxide (DMSO). The absorbance of the solution was then read on an automated plate reader at a wavelength of 570 nm. The percentage of growth compared to the untreated control was transformed into dose–response curves with the GraphPad Prism 5 program and calculated the IC_50_ values. Toxicity test was repeated three times.

### 4.3. Doxorubicin Toxicity

The doxorubicin toxicity was evaluated in absence and in the presence of the SR59230A compound tested at 3 µM and 5 µM concentrations. Cells (10^4^ cells/well) were seeded, in exponential growth phase, and solutions of doxorubicin, or a solution of doxorubicin in combination with the compound, were added to the wells repeated in quadruplicate. Then the plates were incubated at 37 °C for 72 h in a humidified atmosphere with 5% CO_2_. The MTT test was applied as described for intrinsic toxicity and the ability of the SR59230A compound to increase cytotoxicity of doxorubicin was expressed by the RF values obtained as the ratio between the doxorubicin IC_50_ values on K562/DOX cell line in the absence and in the presence of the compound. The same procedure was adopted for the evaluation after 48 h of the reversal activity of resistance in hypoxic environment with 1% O_2_. The cell line was adapted to O_2_ decrease for 24 h. At 48 h, a sample of control cells maintained at 21% O_2_ or 1% O_2_ was collected for the molecular analysis by PCR. Toxicity test was repeated three times.

### 4.4. Reverse Transcription RT-PCR

The expression levels of *MDR1* were analyzed through quantitative PCR (qRT-PCR) using a RotorGene 3000 (Qiagen, Germany) instrument. Primers were purchased from IDT (Germany). An amount of 500 ng of total RNA was retro-transcribed using iScript (Bio-Rad, USA) and amplified with specific primers: *MDR1*, Fw CAGCTATTCGAAGAGTGGGCACAAAC and Rv GCCTCTGCATCAGCTGGACTGTTG. PCR amplification was carried out by SsoAdvancedTM Universal SYBR® Green Supermix (Bio-Rad, USA) according to manual instruction. In the present analysis 18s rRNA was confirmed to be stable and was used as the normalizer Fw CGGCTACCACATCCAAGGAA and Rv GTTATTTTTCGTCACTACCTCCCCGGG. The RT-qPCR was performed using the following procedure: 98 °C for 2 min, 40 cycles of 98 °C for 5 s, 60 °C for 10 s. The program was set to reveal the melting curve of each amplicon from 60 to 95 °C with a read every 0.5 °C. For the *ADRB3* gene the following primers were used: RefSeq Accession No. NC_000008.10, NG_011936.1, NT_167187.1; unique assay ID qHsaCED0047996 (Biorad).

### 4.5. BM and Culture Conditions 

The study was conducted in accordance with the Declaration of Helsinki, and the protocol was approved by the Pediatric Ethics Committee of Regione Toscana (Project identification code “Beta 3 2019 #235/2019”). 

Peripheral Blood (PB) samples of BM blood were collected according to clinical management. BM aspirates were collected from health donors that provided a written informed consent. BMCs were isolated and expanded using α MEM supplemented with 10% heat inactivated FBS, penicillin-streptomycin, 4-(2-hydro-xyethyl)-1-piperazineethanesulfonic acid (HEPES), sodium pyruvate (all from Invitrogen, Mississauga, Ontario, Canada), and 5 ng/mL basic fibroblast growth factor (bFGF or FGF2; from Humanzyme, MedicorpInc., Montreal, Quebec, Canada). BMCs were incubated under standard conditions (at 37 °C in a humidified incubator with 5% CO_2_) at 21% O_2_ or 1% O_2_. 

### 4.6. Flow Cytometric Analysis and Apoptosis Evaluation

K562, KCL22, HEL and HL60 cell lines were cultured in 24-well plates (50,000/well) in a hypoxic or normoxic incubator. In order to evaluate SR59230A toxicity, all cell lines were treated with increasing concentration of SR59230A (1 μM, 3 μM, 6 μM, 8 μM, 10 μM) for 24 h and 48 h.

SR59230A (1 μM, 5 μM, 10 μM, 20 μM, 50 μM) effects on apoptosis were evaluated on BMCs cultured in 24-well plates (50,000/well) in hypoxic (1%) or normoxic (21%) conditions. For the evaluation of β1-, β2-, and β3-adrenoreceptor expression level, K562, HEL and HL60 cell lines were cultured under normoxic and hypoxic conditions for 24 h, and then stained with the anti-β1 (ORB 129489 (PE)), anti-β2 (ORB 15065 (FITC)) (Biorbyt, Caambridge, GB) and anti-β3-AR antibodies. The anti-β3-AR antibody (ab140713) used for cytofluorimetric analysis was obtained from ABCAM and conjugated with the R-Phycoerythrin Conjugation Kit (ab102918, ABCAM). Expression levels were obtained by using a MACSQuant Analyzer 10 flow cytometry. 

Apoptosis was analyzed using FITC Annexin V Apoptosis Detection Kit with Propidium Iodide according to the manufacture’s protocol. The cell lines were washed twice with cold BioLegend’s Cell Staining Buffer and were resuspend in Annexin V Binding Buffer. FITC Annexin V and Propidium Iodide solution were added to cell suspension and further incubated for 15 min at room temperature in the dark; afterwards Annexin V Binding Buffer was added, the cell lines were analyzed by flow cytometry and data were analyzed with Flowlogic Software. Flow cytometry experiments were repeated *3* times.

### 4.7. Colony Formation Assay 

Cord blood cells were obtained from health donors. For the colony formation assay, 100,000/mL of mononucleated cells was plated in p35 mm dishes in Methocult (GF H4434, Voden) and treated with SR59230A (1 μM, 5 μM, 10 μM, 20 μM, 50 μM). After 14 days, the number of BFU-E and CFU-GM were counted. 

### 4.8. Western Blot Analysis 

After cells lysis and quantification, 20 μg of total proteins was loaded on SDS-PAGE followed by WB analysis. PVDF membranes were treated with blocking solution for 1 h at room temperature and then they were incubated overnight at 4 °C with gentle shaking with the following primary antibodies: anti-human HIF-1a #610959 (BD Transduction Laboratories), anti-MDR-1 (E1Y7S) #13978 (Cell Signaling Technology), anti-UCP2 (G-6) sc-390189 and anti-β-Actin (C4) sc-47778 (Santa Cruz Biotechnology). The next day membranes were incubated with specific secondary antibodies for 1 h at room temperature. Chemiluminescent protein revelation was performed using Clarity Western ECL Substrate (Biorad) and the images were acquired through the Chemidoc Imaging System (Biorad®). The Western blot experiments were repeated three times and to verify the application of equal amounts of protein, the intensity of the corresponding protein bands of interest was normalized based on that of the β-actin band for each sample.

### 4.9. Statistical Analysis

Statistical analysis was performed using the GraphPad Prism 6.0 (GraphPad Software, San Diego, CA). Values are presented as mean ± SD. Differences with *P* < 0.05 were considered significant.

To assess normal distribution and homoscedasticity for each quantitative outcome in each group Kolmogorov–Smirnov’s test and Bartlett’s Test was used, respectively. In order to evaluate difference in quantitative outcomes between groups, according to normality and homoscedasticity tests results, ANOVA and posthoc t-test with Bonferroni correction for multiple comparison were used. Posthoc test was performed only if ANOVA analyses were statistically significant.

## 5. Conclusions

**Figure 7:**
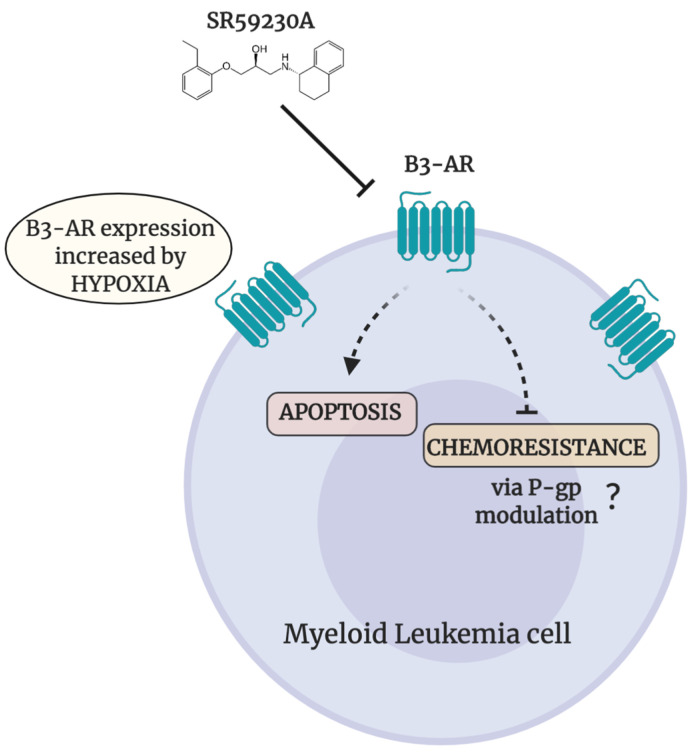
Schematic representation of effects elicited by β3-AR-blockade in myeloid leukemia cell lines. Pharmacological SR59230A β3-AR-blockade induces apoptosis and decreases chemoresistance in myeloid leukemia cell lines. Figure created with BioRender.

In summary, this work offers a new interesting perspective on myeloid leukemias treatment; in particular our data highlight β3-AR as an attractive target to reduce cancer cell survival in myeloid malignancies (Figure 7). Moreover, β3-AR antagonists in combination with MDR chemotherapeutic drugs, could represent a novel strategy to fight and overcome chemoresistance improving clinical outcome and survival of patients affected by myeloid leukemias. However, this work reported preliminary data that need validation through further experimental tests.

## Supplementary Materials

The following are available online at https://www.mdpi.com/1422-0067/21/12/4210/s1. Figure S1. (A) Cell death assessment in myeloid leukemia cell lines silenced for β1-, β2- and β3-ARs in hypoxia and normoxia. Apoptosis evaluation through Annexin V and Propidium Iodide in HEL, K562, and HL60 cell lines transfected with selective siRNA for β1-, β2-, β3-ARs. (B) Average IC_50_ values of doxorubicin and its combination with the SR59230A (referring to Figure 6B). The table also shows the RF (* reversal fold) value calculated from the ratio between the IC_50_ value of doxorubicin in the absence of SR59230A and in the combination with the compound under study. Figure S2. (A) Cell death assessment in myeloid leukemia cell lines treated with L748,337 and Propranolol in hypoxia and normoxia. β-ARs blockers effects on HEL, K562 and HL60 leukemia cell lines. Apoptosis evaluation through Annexin V in HEL, K562 and HL60 cell lines treated with different concentration of L748,337 (5 μM, 10 μM) and Propranolol (5 μM, 10 μM) for 48 h, in normoxia (21% O_2_) and in hypoxia (1% O_2_); Significance was calculated by one-way ANOVA analysis followed by Bonferroni’s post-hoc test. Results are reported as mean ± SD of three independent experiments. n = 3 per group. (* *P* < 0.05, ** *P* < 0.01, *** *P* < 0.001 L748 or Prop vs. Ctrl Normoxia; ° *P* < 0.05, °° *P* < 0.01, °°° *P* < 0.001, °°°° *P* < 0.0001 L748 or Prop vs. Ctrl Hypoxia). (B) β1- β2- and β3-ARs protein expression in myeloid leukemia cell lines in hypoxia and normoxia. Evaluation of β1-AR and β2-AR positive cells in HEL, K562 and HL60 leukemia cell lines in normoxia and hypoxia.

## Abbreviations

HSCs	Hematopoietic Stem Cells
P-gp	P-glycoprotein
ABC	ATP-binding Cassette
MDR1	Multiple Drug Resistance gene
AML	Acute Myeloid Leukemia
β-ARs	beta-Adrenergic receptors
β1-ARs	beta1-adrenoreceptors
β2-ARs	beta2-adrenoreceptors
β3-AR	beta3-adrenoreceptor
BMCs	Bone Marrow Cells
PBMCs	Peripheral Blood Mononuclear Cells
RF	Reversal Fold
FBS	Fetal Bovine Serum
FCS	Fetal Calf Serum
DMSO	Dimethyl Sulfoxide
HEPES	4-(2-hydro-xyethyl)-1-piperazineethanesulfonic acid
bFGF	basic Fibroblast Growth Factor
