# Supplementary material for: β3-Adrenoreceptor Blockade Reduces Hypoxic Myeloid Leukemic Cells Survival and Chemoresistance
Source: Int J Mol Sci. 2020 Jun 12;21(12):4210. doi: 10.3390/ijms21124210 (PMC7352890; doi:10.3390/ijms21124210)
Supplement: Supplementary file 1 [file ijms-21-04210-s001.pdf]

A)

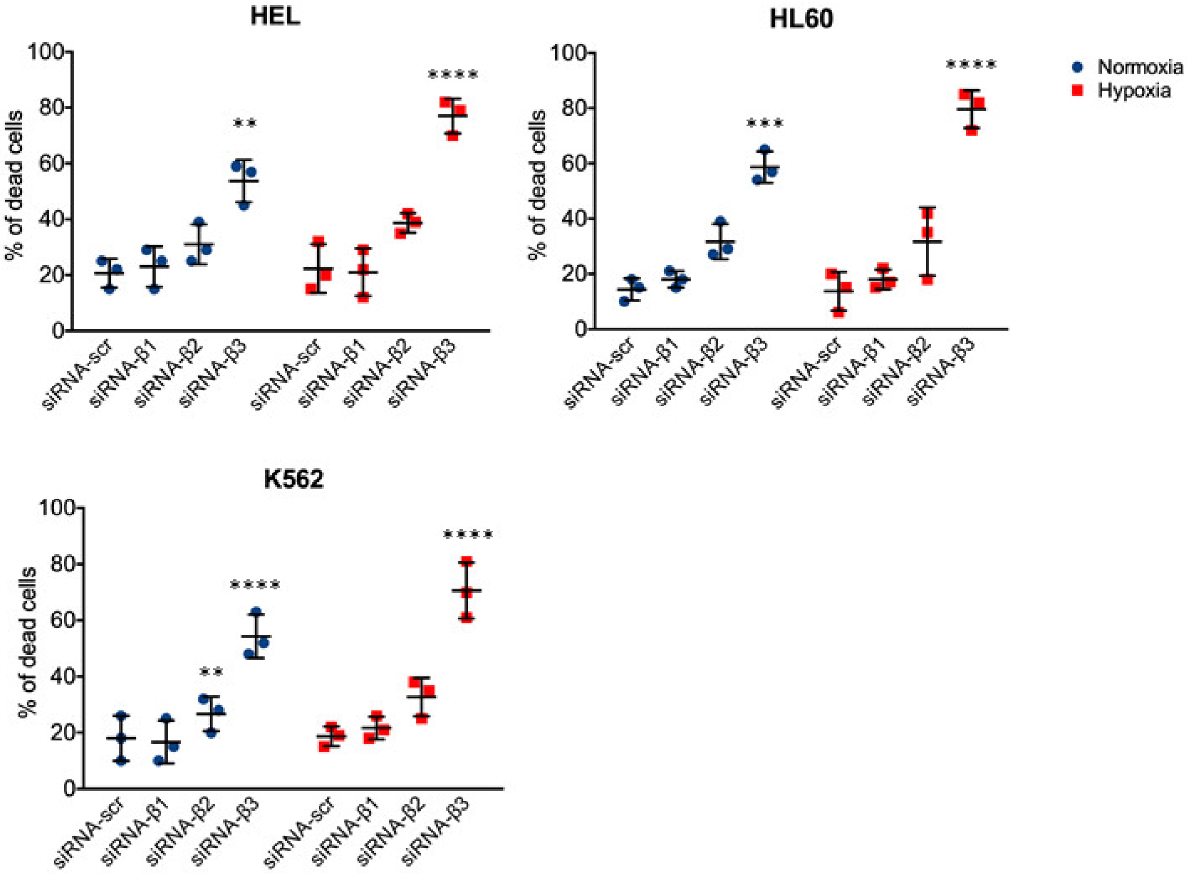

B)

	DOXO	DOXO + SR 3μM	DOXO + SR 5μM
IC50 Normoxia (μM)	0.061	0.089	0.19 (1.4)*
IC50 Hypoxia (μM)	0.27	0.04 (1.55)*	0.019 (14.2)*

Supplementary Figure S1. (**A**) Cell death assessment in myeloid leukemia cell lines silenced for β1-, β2- and β3-ARs in hypoxia and normoxia. Apoptosis evaluation through Annexin V and Propidium Iodide in HEL, K562, and HL60 cell lines transfected with selective siRNA for β1-, β2-, β3-ARs. (**B**) Average IC_{50} values of doxorubicin and its combination with the SR59230A (referring to Figure 6B). The table also shows the RF (*reversal fold) value calculated from the ratio between the IC_{50} value of doxorubicin in the absence of SR59230A and in the combination with the compound under study.

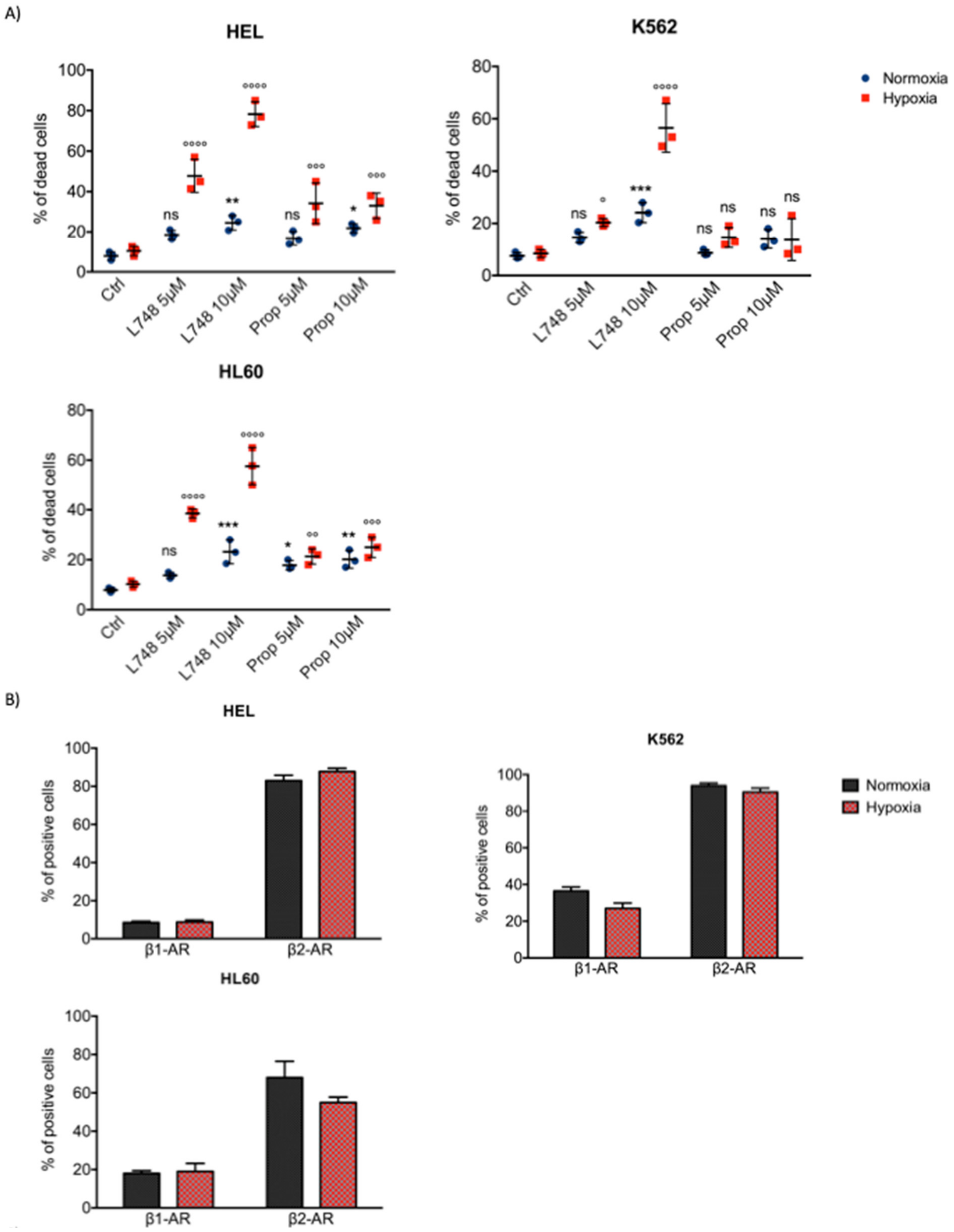

Supplementary Figure S2. (**A**) Cell death assessment in myeloid leukemia cell lines treated with L748,337 and Propranolol in hypoxia and normoxia. β-ARs blockers effects on HEL, K562 and HL60 leukemia cell lines. Apoptosis evaluation through Annexin V in HEL, K562 and HL60 cell lines treated with different concentration of L748,337 (5 μM, 10 μM) and Propranolol (5 μM, 10 μM) for 48 h, in normoxia (21% O_2) and in hypoxia (1% O_2); Significance was calculated by one-way ANOVA analysis followed by Bonferroni's post-hoc test. Results are reported as mean ± SD of three independent experiments. n = 3 per group. (*P < 0.05, **P < 0.01, ***P < 0.001 L748 or Prop vs. Ctrl Normoxia; °P < 0.05, °°P < 0.01, °°°P < 0.001, °°°°P < 0.0001 L748 or Prop vs. Ctrl Hypoxia). (**B**) β1- β2- and β3-ARs protein expression in myeloid leukemia cell lines in hypoxia and

normoxia. Evaluation of β1-AR and β2-AR positive cells in HEL, K562 and HL60 leukemia cell lines in normoxia and hypoxia.
